# Effects of a Nonthermal Atmospheric Pressure Plasma Jet on Human Gingival Fibroblasts for Biomedical Application

**DOI:** 10.1155/2016/2876916

**Published:** 2016-08-11

**Authors:** Jung-Hwan Lee, Kyoung-Nam Kim

**Affiliations:** ^1^Department and Research Institute of Dental Biomaterials and Bioengineering, College of Dentistry, Yonsei University, 50 Yonsei-ro, Seodaemun-gu, Seoul 03722, Republic of Korea; ^2^Institute of Tissue Regeneration Engineering (ITREN), Dankook University, Daeda-ro 119, Dongnam-gu, Cheonan 31116, Republic of Korea; ^3^Department of Dental Hygiene, Kyungdong University, 815 Gyeonhwon-ro, Munmak, Wonju, Gangwondo 24695, Republic of Korea

## Abstract

Nonthermal atmospheric pressure plasma jets (APPJ) have been developed and applied in biomedical research as a cancer treatment or bacterial sterilization. However, the drawback of APPJ on normal oral cells during plasma treatment and underlying cell death mechanisms have not been studied and clearly explained, although there is known to be an influence from reactive oxygen species (ROS). Hence, this study investigates whether and how a nonthermal atmospheric pressure air plasma jet kills human normal gingival cells using immortalized human gingival fibroblasts (hTERT-hNOF cells). In this study, a set of physicochemical or biological methods were used to illuminate the killing mechanisms. It was found that ROS were induced intracellularly without a breakdown of the cell wall and apoptosis was involved in cell death when an air APPJ treatment was performed on the cells directly without media; the air treatment only supported a detachment of the cells without increase of ROS. It was also revealed that a correlation between intracellular ROS concentration and cells viability existed. These results indicated that the direct air APPJ treatment possibly raises safety issue to normal tissue and thereby APPJ application in biomedical field needs more* in vitro* and* in vivo* study to optimize it.

## 1. Introduction

Killing cancer cells and eradicating harmful microorganisms in the environment are both important aims [1]. Current strategies for cancer treatment are based on the removal of cancer cells through surgery. To reduce the risk of recurrence by residual cancer cells, an adjuvant treatment is used that consists of radio-radiation, chemotherapy, or proton beam and magnetic nanoparticles [2–4]. However, adjuvant treatments such as chemotherapeutic drugs have side effects as they may also kill normal cells or mistakenly target noncancerous cells due to their high proliferation. Therefore, the development of a novel therapeutic method for killing target cells is strongly needed. As a novel therapeutic technique, nonthermal atmospheric pressure plasma jets (APPJ) have been considered in recent years due to their many advantages, which include operation at atmospheric pressure, application to target sites without heat-induced damage, and cost-effective, portable systems [5, 6]. However, the side effect of APPJ on normal oral cells during plasma treatment for cancer therapy has not been studied to the author's knowledge.

APPJ therapy within many biomedical applications could modulate the various biological processes of cells and thus can be used as a substitute for conventional cancer therapeutics or sterilization [1, 7–9]. In order to make APPJ therapy more effective in killing the target cells, a study of cell death mechanisms is essential. First, plasma-irradiated cells lose their ability to attach and incurred cellular changes, which clearly shows the response of mammalian cells to plasma species [10]. Second, it has been reported that plasma therapy can induce apoptosis by plasma radicals directly [11]. Furthermore, plasma-induced apoptosis has been clearly demonstrated in a recent paper that investigated cellular signaling related to an apoptotic process [12, 13]. Recent research suggests that reactive oxygen species and reactive nitrogen species (ROS/RNS) are significant and perhaps even central actors in the actions of antimicrobial and cancer therapies [14, 15]. However, the cell membrane damage by APPJ as a role for cell death and correlation between quantity of intracellular ROS and cell viability have not been clearly explained other than the influence of ROS induced apoptosis.

To evaluate the side effect of APPJ treatment in oral cavity and cell death mechanism of human cells by APPJ treatment, immortalized human normal gingival fibroblasts with human telomerase reverse transcriptase gene transfection (hTERT-hNOF cells) were selected for this study. Using human cell lines derived from normal oral tissues would be considered more appropriate choice for evaluating side effect of normal cells by APPJ treatment for cancer treatment, compared to malignant cell lines (e.g., L929, Saos-2). The study was undertaken using human normal gingival fibroblasts (hNOF) in situ in order to evaluate biocompatibility of titanium dental implant in contact with the mucosal gingival [16]. However, such hNOF cannot be consistently used for evaluating cytotoxicity of dental products, due to the limited lifespan in serial culture and the genomic instability in later passages [17, 18]. It was studied that the cytotoxicity response of hTERT-hNOF cells is significantly similar to human normal gingival fibroblasts (hNOF) and more sensitive compared to L929 cells with genomic stability even at the later passages [19].

Hence, the aim of this study is to reveal the effect of APPJ on immortalized human gingival fibroblasts during plasma treatment and the normal human cell death mechanism by direct APPJ treatment, in terms of the plasma etching phenomenon, cell detachment, cell wall destruction, and ROS accumulation.

## 2. Experimental Set-Up

### 2.1. Plasma Source

The treatment consisted of an air plasma jet (Kwangwoon University, Korea) [20] with a compressed air flow rate of 3 L/min. Briefly, the plasma jet components consisted of a steel inner electrode having 1.2 mm depth, 0.2 mm thickness, and 3.2 mm depth of quartz as a dielectric. The hole in the outer electrode was 0.7 mm. An oscilloscope (Lecroy Wave Runner 64xi (10 GS/s)), voltage probe (Tektronix P6015A (1000 : 1)), and Neon Trans (PNP-1000, max output voltage 15 kV, current 13 mA) were included in the plasma jet equipment. The plasma treatment time ranged from 5 seconds to 4 minutes.

### 2.2. Determination of the Etching Effect of APPJ

To determine the etching effect of the air APPJ treatment, a polystyrene plate surface was analyzed by an optical 3D surface measurement profile (Contour GT-X3 BASE, Bruker, Bremen, Germany) after treatment with air and the air APPJ for 5 minutes. Each specimen was visualized in 2D and 3D images and was further analyzed by the Ra value in order to determine the difference in roughness between groups. The scan length and backscan were 5 and 15 *μ*m in the vertical scanning interferometry mode, respectively. Magnification of the objective and multiplier was 2.5x and 0.55x, respectively.

### 2.3. Determination of the Chemical Composition of Cell Debris

After hTERT-hNOF cells (1.5 mL of 1 × 10^5^ cells/mL) were incubated in Dulbecco's Modified Eagles Medium/F-12 (DMEM/F-12 3 : 1, Welgene, Daegu, Korea) supplemented with 10% fetal bovine serum (FBS, Gibco, Grand Island, NY) and 1% antibiotic-antimycotic (Gibco, BRL, USA) for 3 days, it was washed twice by phosphate buffer solution (PBS, Welgene) to remove residual media. The air or air APPJ treatment was performed on adherent cells after discarding the PBS. Next, 200 *μ*L of cell lysis buffer (Cell signaling Technology, Beverly, MA) was added, and the cells were incubated at 4°C for 10 minutes. After the cells were manually scraped into a tube using a cell scraper, they were briefly sonicated and centrifuged for 20 minutes at 15000 ×g at 4°C. The pellets including inclusion bodies, which are nuclear or cytoplasmic aggregates (proteins) of stainable substances, without DNA, RNA, and soluble protein were gathered and washed twice by PBS after discarding the supernatant. A freeze dryer (Bondiro, Ilshin, Kyungki-do, Korea) was used for one day at −48°C with 7 mTorr to dry the pellets. X-ray photoelectron spectra (XPS, Thermo Scientific, UK) were obtained using K-alpha. The X-rays were irradiated on the pellets of cells. C1s at 284.8 eV were used as a reference, and C, O, N, P, and Na were detected in detail.

### 2.4. Determination of Intracellular Accumulation of ROS

To monitor the intracellular accumulation of ROS, the cell-permeable fluorescent probe 2′, 7′-dichlorodihydrofluorescein diacetate (DCFH-DA, Cell Biolabs Inc., San Diego, CA) was used. DCFH-DA was oxidized to highly fluorescent 2′, 7′-dichlorodihydrofluorescein (DCF) by ROS after diffusion into the cells. The fluorescence intensity is known to be proportional to the ROS levels within the cell cytosol [21]. Procedures were followed according to the manufacturer's protocol. Briefly, 100 *μ*L of 1x DCFH-DA/media solution was added to each well of a 96-well black plate, which had 3-day cultured hTERT-hNOF cells (cell seeding amount is 100 *μ*L of 1 × 10^5^ cells/mL per well). After the plate was incubated at 37°C for 30 minutes, the solution was removed, and each well was washed twice by DPBS. After air or air APPJ treatment was performed on DCFH-DA loaded hTERT-hNOF cells without any intermediate solution, a fluorescent microplate reader was used to detect the fluorescence, using excitation and emission wavelengths of 480 nm and 530 nm, respectively, after 20 minutes of further incubation with supplemented media. After obtaining the standard curve between the DCF concentration and the relative fluorescent unit (RFU), a correlation was calculated between the cell viability and the concentration of DCF, as the quantity of intracellular ROS. After preparing a 1 : 10 dilution series of DCF standards in the concentration range of 0 nM–10 *μ*M, by diluting the 1 mM DCF stock in the cell culture media, 75 *μ*L of each standard and cell lysis buffer was transferred to a 96-well plate and a fluorescence measurement was performed at the above wavelengths.

### 2.5. Cell Viability Test

Cell viability was determined using a cell viability assay kit which consumes WST salt (Ez-Cytox, Daeillab, Korea). The amount of yellow formazan dye that is generated from the cells is known to be directly proportional to the number of viable cells, because WST salt changes into a highly water soluble dye by the mitochondrial NADH-dehydrogenase activity of live cells. The immortalized human gingival fibroblasts, hTERT-hNOF cells, were established by transfecting human normal gingival fibroblasts with human telomerase reverse transcriptase. This preserved the phenotypical characteristics, replicative potential, and biological properties of human normal gingival fibroblasts [19]. hTERT-hNOF cells were cultured in supplemented DMEM/F-12 3 : 1 media by the way that was previously described in Section 2.3. The cells were maintained in a humidified incubator at 37°C, in an atmosphere containing 5% CO_2_. After hTERT-hNOF cells (500 *μ*L of 1 × 10^5^ cells/mL) were seeded on each well of a 12-well polystyrene plate (SPL, Pocheon, Gyeonggi-do, Korea), they were incubated for 3 days without change of media prior to treatment. The air or air APPJ treatment was performed without any liquid on adherent cells after discarding the media and washing the cells with PBS. The absorbance was measured in a spectrophotometer at a wavelength of 450 nm after washing by PBS, and 3 h after adding 50 *μ*L (10% of supplemented media) of WST solution. The results were expressed as a percentage of the values obtained from an untreated specimen as a control group.

### 2.6. Determination of Cell Detachment and the Morphology Change

After air APPJ treatment of hTERT-hNOF cells in a way of 2.5. and 3 hr further incubation in supplemented media, each sample was visualized as a bright field image of cells using a Juli*™* fluorescent cell analyzer (Digital Bio, Seoul, Korea). The samples were prepared by following the procedures for a scanning electron microscopy image. The specimens were fixed with 2% glutaraldehyde and 2% paraformaldehyde with 0.5% CaCl_2_ in 0.1 M pH 7.4 phosphate buffer for 2 h and washed three times for 30 min in 0.1 M PB. The samples were postfixed with 1% OsO_4_ dissolved in 0.1 M PB for 2 h, dehydrated in an ascending gradual series (50~100%) of ethanol, followed by T butyl alcohol, and then subjected to a freeze dryer (Hitachi, Japan). They were then coated with gold using an ion sputter (IB-3 Eiko Japan) of 6 mA for 6 min and examined and photographed with a FE-SEM (S-800, Hitachi, Japan) at the acceleration voltage of 20 kV.

### 2.7. Annexin V-PI Cytofluorimetric Analyses

Cell apoptosis and necrosis were analyzed with the annexin V-fluoroisothiocyanate apoptosis detection kit (BD Biosciences Pharmingen, CA, USA) following the manufacturer's instructions. hTERT-hNOF cells incubated for 3 days as described in Section 2.5. were treated with 15-second air or air APPJ. After treatment, 3 hr of incubation was performed in supplemented media before analysis. The samples were analyzed by flow cytometry (FC 500, Beckman Coulter, CA, USA) with 10,000 cells per each running.

### 2.8. Statistical Analysis

One-way ANOVA and Tukey's test were used to analyze the differences between groups. The significance level was set at 95% (*p* = 0.05). The Pearson correlation test was performed between groups at the 95% level.

## 3. Results and Discussion

**Figure 1:**
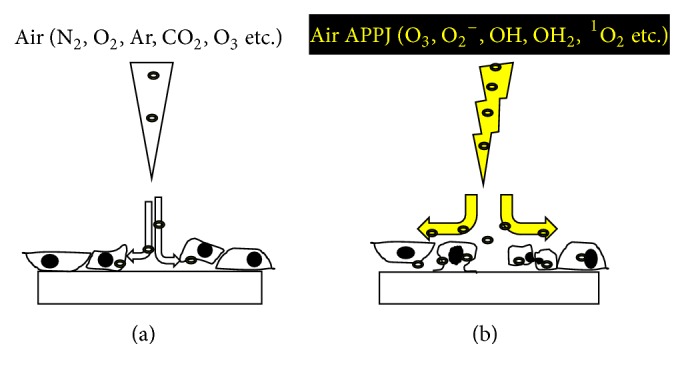
Schematic diagram illustrating the main hypotheses explaining the cell death mechanism: (a) by direct air through detachment and (b) by air APPJ treatment on cells with an expression of apoptosis-like or split morphology by intracellular ROS.

Three possible cell responses to the plasma treatment have been reported: programmed cell death (apoptosis), temporary loss of cell adhesion, and necrosis [7]. Since the cell response to air APPJ produced in ambient air was strongly affected by the air flow, the main hypotheses postulated to explain the cell death mechanism by direct air and by air APPJ treatment on cells are explained in the schematic diagram in Figure 1. With the APPJ treatment, apoptosis and loss of cell adhesion possibly occurred, while the air treatment only induced the detachment of cells on the surfaces.

**Figure 2:**
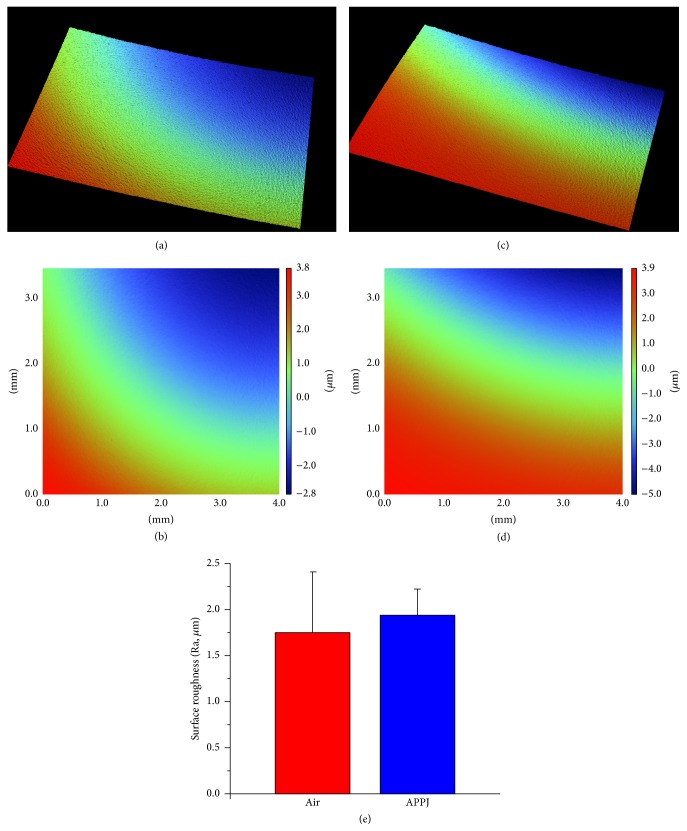
(a), (c) 3D and (b), (d) 2D surface roughness images of an (a), (b) air or (c), (d) air APPJ treated polystyrene plate, and (e) a comparison graph of the Ra value (*n* = 5).

Among various plasma effects—such as etching, heat damage, and metallization or polymerization of single materials on the biomaterial surfaces—measurements of the etching effect in terms of surface roughness had the greatest priority, as heat damage from APPJ was excluded in the device developmental stage and another material was not used in this study [20, 22, 23].  Figure 2(e) shows that a surface roughness change (Ra) after APPJ treatment was not statistically observed (air: 1.94 ± 0.28 *μ*m, air APPJ: 1.74 ± 0.66 *μ*m). Although each treatment had a different mean value about the surface roughness, the 3D and 2D surface roughness images in Figures 2(a)–2(d) confirmed that there was no visibly etched morphology on the surface. This lack may be advantageous for creating a warranted plasma effect on cells without physical ablation.

**Figure 3:**
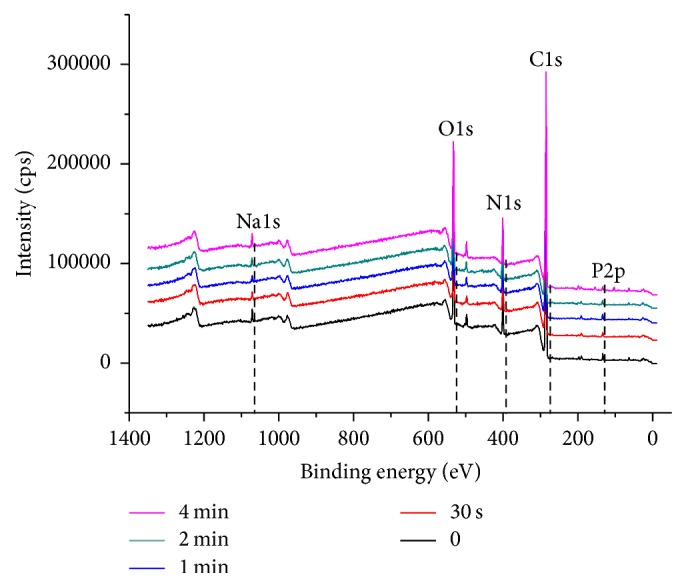
XPS analysis of cell debris after the air APPJ treatment on the hTERT-hNOF cells. P2p (133.2 eV), C1s (285.1 eV), N1s (399.9 eV), O1s (531.9 eV), and Na1s (1071.1 eV) were majorly detected.

An XPS analysis of cell debris, consisting of a cell wall and organelle, is shown in Figure 3. Because there was strong relationship between the N/P atomic concentration ratio of the inclusion bodies, mostly composed of insoluble lipid cell membrane, and the electrophoretic mobility of them, XPS was applied to investigate any change of inclusion bodies to investigate damage of insoluble lipid cell membrane, which was important for cell membrane integrity [24]. In this study, the significant change of N/P ratio as atomic percentage was not found in air APPJ treatment groups (5.8~6.2) compared to nontreated control (6.0 ± 0.4). Therefore, the composition change in the insoluble cell lipid membrane consisting of cell membrane was not observed significantly, which means that plasma radicals rarely damage the cell wall directly.

**Figure 4:**
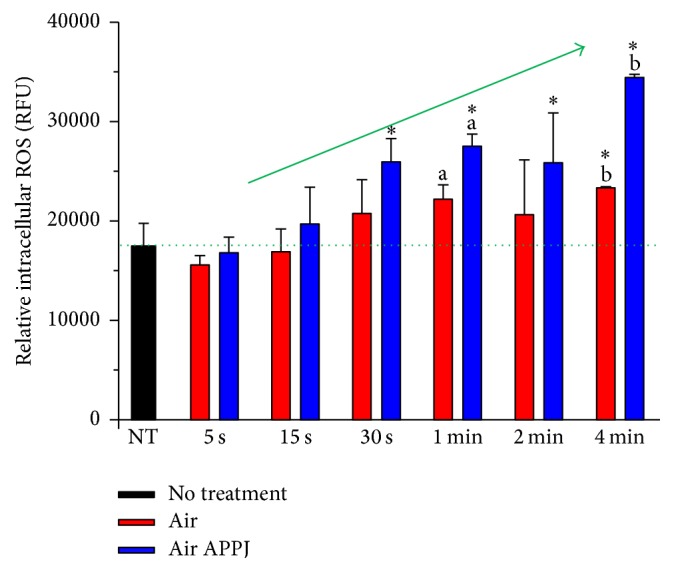
Intracellular reactive oxygen species (ROS) after air or air APPJ treatment on hTERT-hNOF cells. ROS was measured 20 minutes later after treatment. Statistic difference is shown between same letters in same treatment time. Asterisk indicates significant difference compared to NT (*n* = 5, *p* < 0.05).

The quantity of relative intracellular ROS according to the treatment in the hTERT-hNOF cells was investigated after 20 minutes from the treatment time to calculate quantity of immediately generated ROS.  Figure 4 shows that the quantity of intracellular ROS by air or air APPJ treatment on hTERT-hNOF cells increased with an increase in the treatment time; furthermore, this quantity was statistically different between air and air APPJ in the 1 m and 4 m treatment groups (*p* < 0.05). There have been some reports that ROS exists inside the cell membrane. ROS may bind to the cell membrane receptors and the activated intracellular signaling pathways that modify the intracellular ROS concentration [25]. Alternatively, ROS may move across the cell membrane by active transport across the bilayer, through a transient opening of pores in the membrane or in calcium channels [2, 26]. This study used a cell permeable fluorescent probe (DCFH-DA) to monitor the intracellular accumulation of ROS by both of these options. DCFH-DA can only be oxidized to highly fluorescent DCF by intracellular ROS after it is deacetylated to DCFH by cellular esterases. In addition, suctioning of the cell media prior to treatment was performed to minimize the interactions between ROS and the cell media, including amino acids and proteins, and also to minimize the production of long-lived reactive organic hydroperoxides in the media. This in turn reduced the lipid peroxidation and cell membrane damage by plasma-media interaction [27]. Therefore, the fluorescence intensity by DCF has been used to detect the quantity of selective ROS that is induced by the plasma radicals themselves, and not by the plasma-media interaction.

**Figure 5:**
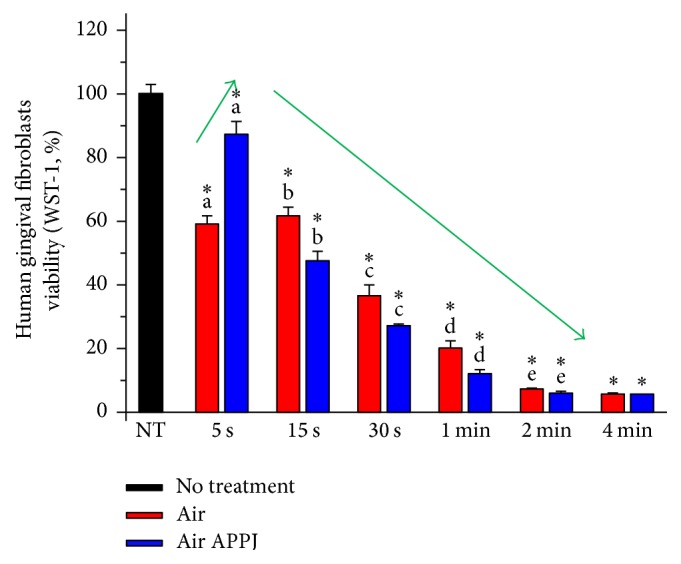
hTERT-hNOF cells viability after air or air APPJ treatment. Cell viability was measured 3 hr later after treatment. Statistic difference is shown between same letters in same treatment time. Asterisk indicates significant difference compared to NT (*n* = 4, *p* < 0.05).

To reveal the effect of APPJ treatment on human normal cells and correlation between cell viability and the quantity of intracellular ROS, a cell viability test was first performed. It is known that plasma induces a time dependent cell death in several cells (i.e., human cancer cells and canine blood cells) [28, 29]. The cancer cells were easily eliminated by a few-second treatment of direct air APPJ in previous study (no results); in extreme condition up to 4-minute treatment was performed for evaluating side effect of APPJ treatment on human oral gingival fibroblasts in this study. However, the effect of air APPJ on human oral gingival fibroblasts, in case of APPJ application in oral cavity, has been rarely studied. To precisely evaluate the intracellular ROS effects by APPJ, the air treatment was used as a control group.  Figure 5 shows that hTERT-hNOF cells viability after air or air APPJ treatment decreased as the treatment time increased, except for the 5-second treatment. The results for the 5-second short treatment show that the drying effects of the air flow reduce cell viability, while the plasma radicals induce a moderate increase in viability. In terms of the evaluation of the APPJ effects on cells, the air APPJ treatment had statistically lower hTERT-hNOF cells viability compared to the air treatment, except for the 5-second and 4-minute treatment (*p* < 0.05). The correlation between the air APPJ treated cell viability and the calculated DCM concentrations of treated cells are described in Table 1. The quantities of calculated DCM in the air APPJ and air treated cells are correlated with the cell viability of air APPJ-treated cells at the 0.05 level, except for that of air subtracted from air APPJ. The above correlation shows the quantity of intracellular ROS induced ablation of hTERT-hNOF cells.

Since ultraviolet (UV) radiation also has the potential to reduce cell viability, UV effects were eliminated from the previous study. No species were detected under 300 nm, at which the strongest effects of UV occur in biological systems for the ablation of bacteria and organic cells; also, very few species were observed near UV light (300–400 nm) in the exact same air APPJ system, as detected using optical emission spectrum analysis [30].

**Figure 6:**
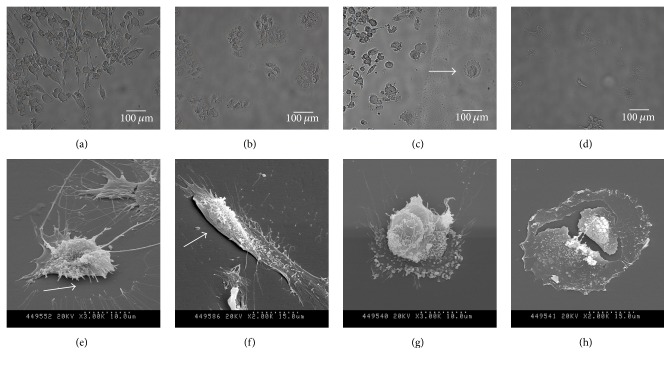
Bright image of hTERT-hNOF cells after 15 second (a and b) air and (c and d) air APPJ treatment, and SEM image after 15-second (e and f) air and (g and h) air APPJ treatment. After treatment, 3 hr of incubation was performed in supplemented media before observation.

A comparison of cell phenotypes after air treatment and after air APPJ treatment (Figure 6) clearly shows that the air APPJ treatment causes apoptosis-related morphological changes in hTERT-hNOF cells compared to the air treatment. In Figure 6(a), the ablation area of cells by air flow clearly appeared with detached cells (Figure 6(b)). However, the ablation of cells by air APPJ obviously appeared (Figure 6(c)) with apoptosis (indicated by arrow) and a cell shrinkage or condensation (Figure 6(d)) morphology. This apoptosis phenomenon was even more evident in Figure 6(g), where typical apoptosis morphology—surface blebbing which describes bubbling of the plasma membrane—was mostly observed. However, it was rarely observed that the plasma also has a direct physical impact on the cells to make them “burst.” Otherwise, the loss of cell attachment and the breakage of the outer actin filament were observed after the air flow treatment in Figures 6(e) and 6(f). Mammalian cell death consists of three morphologically distinct types: apoptosis, necrosis, and autophagic cell death (ACD) [31]. There have been no reports to date that mammalian cells can be killed via ACD* in vivo*, and apoptosis and necrosis are commonly studied under various physiological conditions. Between the remaining two cell death types, apoptosis is the preferable choice due to a minimal inflammatory reaction by the “programmed cell death mechanism” compared to the large inflammatory reaction of necrosis from dead cells and the innate immune system [11, 32]. The above evaluation of cell morphology showed that apoptosis possibly occurred after air APPJ treatment on human gingival fibroblasts, while necrosis rarely occurred, and the air treatment only supported detachment of the cells.

**Figure 7:**
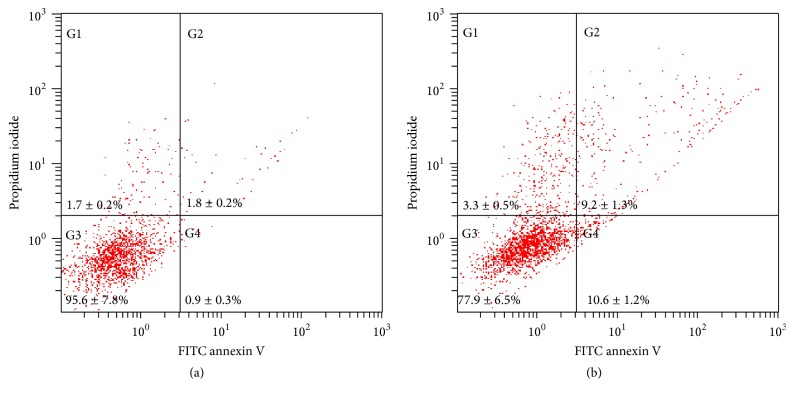
Annexin V-PI cytofluorimetric analyses were performed to observe apoptosis. hTERT-hNOF cells were treated with 15-second (a) air or (b) air APPJ. After treatment, 3 hr of incubation was performed in supplemented media before analysis. 10,000 cells were counted per each running.

Annexin V-PI cytofluorimetric analyses were performed to evaluate whether the cell viability loss was due to induction of apoptosis or necrosis. According to the results, ~20% of cells were undergoing early or late apoptosis in air APPJ treatment while ~3% of cells were necrosis (Figure 7(b)), which was also found in other APPJ treatment against oral cells. The annexin V-PI staining confirmed more apoptotic death in air APPJ treatment [12]. In contrast, air treatment showed most of the cells were alive with only few % of apoptotic or necrotic cells along with the results from direct APPJ treated oral cells [33] (Figure 7(a)). Therefore, the direct air APPJ treatment on human gingival fibroblasts possibly induced warranted cell ablation with minimal necrosis induced inflammatory reaction.

In this study, we used immortalized normal oral cells not cancer cells. Therefore, it cannot be assumed exactly whether killing selectivity among cancer cells and normal gingival cells was obtained. However, according to previous studies [12, 13, 34], nonthermal atmospheric pressure plasma is able to get selective killing effect between normal gingival cells and cancer cells due to sensitivity against reactive species including ROS and difference of involved biological pathway to deal with plasma generated reactive species. Further study is necessary to investigate application of air APPJ treatment for cancer treatment.

## 4. Conclusions

In this study, air APPJ was used to study whether a nonthermal atmospheric pressure air plasma jet kills immortalized human gingival fibroblasts during treatment and to reveal the cell death mechanism by direct treatment on immortalized human gingival fibroblasts, in terms of the plasma etching phenomenon, cell detachment, cell wall destruction, and ROS accumulation. Air APPJ possibly incurs death of human gingival fibroblasts during treatment according to increase of treatment time over 10 seconds without etching damage and destruction of cell wall by direct treatment, as seen by 3D image and XPS analysis. With a correlation between intracellular ROS concentration and hTERT-hNOF cells viability, it was found that the ablation of cells by air APPJ was caused by the interaction between plasma radicals and intracellular reaction. Bright and SEM images with annexin V-PI assays confirmed that, in most cases, direct air APPJ treatment on cells induced apoptosis without a breakdown of the cell membrane, while the air treatment supported the detachment of cells without further apoptosis. These results indicated that the direct air APPJ treatment possibly decrease the number of human gingival fibroblasts according to an increase of treatment time by intracellular ROS activated apoptosis and thereby safety issue to normal tissue will remain for APPJ application.

## Figures and Tables

**Table 1:** Pearson correlation between air APPJ-treated cell viability and calculated DCM concentrations of treated cells.

	Air APPJ-air	Air APPJ	Air
Pearson correlation	0.784	0.873^*∗*^	0.920^*∗*^
Significance	0.065	0.023	0.009

^*∗*^Correlation is significant at the 0.05 level.

## References

[1] Uhm H. S., Choi E. H., Cho G. S., Hong Y. C. (2012). Sterilization of microbes by using various plasma jets. *Journal of the Korean Physical Society*.

[2] Sensenig R., Kalghatgi S., Cerchar E. (2011). Non-thermal plasma induces apoptosis in melanoma cells via production of intracellular reactive oxygen species. *Annals of Biomedical Engineering*.

[3] Lee K.-S., Shin J.-S., Nam K.-S., Shon Y.-H. (2012). Anti-angiogenic activity in metastasis of human breast cancer cells irradiated by a proton beam. *Journal of the Korean Physical Society*.

[4] Lee K.-J., An J.-H., Shin J.-S., Kim D.-H., Yoo H.-S., Cho C.-K. (2011). Biostability of *γ*-Fe_2_O_3_ nano particles evaluated using an in vitro cytotoxicity assays on various tumor cell lines. *Current Applied Physics*.

[5] McCombs G. B., Darby M. L. (2010). New discoveries and directions for medical, dental and dental hygiene research: low temperature atmospheric pressure plasma. *International Journal of Dental Hygiene*.

[6] Deng S. X., Cheng C., Ni G. H., Meng Y. D., Chen H. (2010). *Bacillus subtilis* devitalization mechanism of atmosphere pressure plasma jet. *Current Applied Physics*.

[7] Kim W., Woo K.-C., Kim G.-C., Kim K.-T. (2011). Nonthermal-plasma-mediated animal cell death. *Journal of Physics D: Applied Physics*.

[8] Gweon B., Kim D. B., Moon S. Y., Choe W. (2009). *Escherichia coli* deactivation study controlling the atmospheric pressure plasma discharge conditions. *Current Applied Physics*.

[9] Attri P., Venkatesu P., Kaushik N. (2012). Effects of atmospheric-pressure non-thermal plasma jets on enzyme solutions. *Journal of the Korean Physical Society*.

[10] Kieft I. E., Broers J. L. V., Caubet-Hilloutou V., Slaaf D. W., Ramaekers F. C. S., Stoffels E. (2004). Electric discharge plasmas influence attachment of cultured CHO K1 cells. *Bioelectromagnetics*.

[11] Fridman G., Shereshevsky A., Jost M. M. (2007). Floating electrode dielectric barrier discharge plasma in air promoting apoptotic behavior in Melanoma skin cancer cell lines. *Plasma Chemistry and Plasma Processing*.

[12] Lee J., Om J., Kim Y. (2016). Selective killing effects of cold atmospheric pressure plasma with NO induced dysfunction of epidermal growth factor receptor in oral squamous cell carcinoma. *PLoS ONE*.

[13] Weiss M., Gümbel D., Hanschmann E.-M. (2015). Cold atmospheric plasma treatment induces anti-proliferative effects in prostate cancer cells by redox and apoptotic signaling pathways. *PLoS ONE*.

[14] Graves D. B. (2012). The emerging role of reactive oxygen and nitrogen species in redox biology and some implications for plasma applications to medicine and biology. *Journal of Physics D: Applied Physics*.

[15] Conway G. E., Casey A., Milosavljevic V. (2016). Non-thermal atmospheric plasma induces ROS-independent cell death in U373MG glioma cells and augments the cytotoxicity of temozolomide. *British Journal of Cancer*.

[16] Brunot C., Grosgogeat B., Picart C., Lagneau C., Jaffrezic-Renault N., Ponsonnet L. (2008). Response of fibroblast activity and polyelectrolyte multilayer films coating titanium. *Dental Materials*.

[17] Enoch S., Wall I., Peake M. (2009). Increased oral fibroblast lifespan is telomerase-independent. *Journal of Dental Research*.

[18] Shelton D. N., Chang E., Whittier P. S., Choi D., Funk W. D. (1999). Microarray analysis of replicative senescence. *Current Biology*.

[19] Illeperuma R. P., Park Y. J., Kim J. M. (2012). Immortalized gingival fibroblasts as a cytotoxicity test model for dental materials. *Journal of Materials Science: Materials in Medicine*.

[20] Hong Y. C., Kang W. S., Hong Y. B., Yi W. J., Uhm H. S. (2009). Atmospheric pressure air-plasma jet evolved from microdischarges: eradication of *E. coli* with the jet. *Physics of Plasmas*.

[21] Bass D. A., Parce J. W., Dechatelet L. R., Szejda P., Seeds M. C., Thomas M. (1983). Flow cytometric studies of oxidative product formation by neutrophils: a graded response to membrane stimulation. *The Journal of Immunology*.

[22] Kim D., Lee S., Hwang W. (2012). Complete wetting characteristics of micro/nano dual-scale surface by plasma etching to give nanohoneycomb structure. *Current Applied Physics*.

[23] Cho S.-J., Shrestha S. P., Boo J.-H. (2011). Surface treatment for Cu metallization on polyimide film by atmospheric pressure dielectric barrier discharge plasma system. *Current Applied Physics*.

[24] Amory D. E., Mozes N., Hermesse M. P., Leonard A. J., Rouxhet P. G. (1988). Chemical analysis of the surface of microorganisms by X-ray photoelectron spectroscopy. *FEMS Microbiology Letters*.

[25] Kawiak A., Piosik J., Stasilojc G. (2007). Induction of apoptosis by plumbagin through reactive oxygen species-mediated inhibition of topoisomerase II. *Toxicology and Applied Pharmacology*.

[26] Stoffels E., Kieft I. E., Sladek R. E. J., Van Den Bedem L. J. M., Van Der Laan E. P., Steinbuch M. (2006). Plasma needle for in vivo medical treatment: recent developments and perspectives. *Plasma Sources Science and Technology*.

[27] Gebicki S., Gebicki J. M. (1993). Formation of peroxides in amino-acids and proteins exposed to oxygen free-radicals. *Biochemical Journal*.

[28] Kim C.-H., Bahn J. H., Lee S.-H. (2010). Induction of cell growth arrest by atmospheric non-thermal plasma in colorectal cancer cells. *The Journal of Biotechnology*.

[29] Baik K. Y., Kim Y. H., Hur E.-H. (2012). Selective toxicity on canine blood cells by using atmospheric-pressure plasma jets. *Journal of the Korean Physical Society*.

[30] Husman A. M. D. R., Bijkerk P., Lodder W. (2004). Calicivirus inactivation by nonionizing (253.7-nanometer-wavelength [UV]) and ionizing (gamma) radiation. *Applied and Environmental Microbiology*.

[31] Kroemer G., Levine B. (2008). Autophagic cell death: the story of a misnomer. *Nature Reviews Molecular Cell Biology*.

[32] Zitvogel L., Casares N., Péquignot M. O., Chaput N., Albert M. L., Kroemer G. (2004). Immune response against dying tumor cells. *Advances in Immunology*.

[33] Lee J.-H., Choi E.-H., Kim K.-M., Kim K.-N. (2016). Effect of non-thermal air atmospheric pressure plasma jet treatment on gingival wound healing. *Journal of Physics D: Applied Physics*.

[34] Ma Y., Ha C. S., Hwang S. W. (2014). Non-thermal atmospheric pressure plasma preferentially induces apoptosis in p53-mutated cancer cells by activating ROS stress-response pathways. *PLoS ONE*.

